# Investigation of the effects of two exercise protocols on behavioral and biochemical changes in ovariectomized rats exposed to single prolonged stress

**DOI:** 10.1016/j.ibneur.2026.09.003

**Published:** 2026-09-05

**Authors:** Arvin Amiri, Ali Shushtari, Maryam Janbazi, Seyed Parsa Seyedpour, Seyedeh rose Jamali, Sakineh Shafia, Kobra Akhoundzadeh

**Affiliations:** a Department of veterinary medicine, Faculty of veterinary medicine, Babol Branch Islamic Azad University, Babol, Iran; b Faculty of Medicine, Mazandaran University of Medical Sciences, Sari, Iran; c Bachelor of Medical Engineering, Department of Medical Engineering and Electrical Engineering, Hadaf Institute of Higher Education, Sari, Iran; d Department of Biology, Faculty of biotechnology, Babol Branch Islamic Azad University, Babol, Iran; e Department of Physiology, Immunogenetics research center, Mazandaran university of Medical sciences, Sari, Iran; f Department of Physiology, Qom University of Medical Sciences, Qom, Iran

**Keywords:** PTSD, SPS, Anxiety-Like Behavior, Voluntary Exercise, Forced Exercise

## Abstract

Our previous study demonstrated that ovariectomy exacerbated stress-induced impairments in rats exposed to single prolonged stress (SPS) and that the treadmill exercise protocol used in that study improved the associated cellular responses. However, this protocol did not alleviate all anxiety-like behaviors. Therefore, the present study aimed to determine whether the voluntary wheel-running protocol used in this study would produce greater improvements in anxiety-like behavior than the previously evaluated treadmill exercise protocol. Female adult Wistar rats were randomly allocated to the control (CON), sham, and ovariectomized (OVX) groups. Following PTSD induction, animals within each group were randomly assigned to sedentary (SED), treadmill running (TR), or wheel running (RW) groups. Twenty days after ovariectomy, PTSD was induced by a Single Prolonged Stress (SPS) model. The exercise began on day 32 and continued for 4 weeks. On the 61st day, Open Field Test (OFT), Object Location Memory Task (OLMT), corticosterone, and neurotrophins levels were assessed. p < 0.05 was considered a significant level. The findings revealed significant differences between the OVX/SPS/TR and OVX/SPS/RW groups in hippocampal and prefrontal cortical BDNF and IGF-1 levels. Significant differences were also observed between the OVX/SPS/SED group and both the OVX/SPS/TR and OVX/SPS/RW groups in the OLMT and OFT. The wheel-running protocol produced greater therapeutic benefits against SPS-induced impairments that were exacerbated by ovariectomy. Based on the results of the present study, the exercise protocol appears to be an important factor in achieving targeted therapeutic outcomes in an animal model of PTSD. Exercise-induced stress load, as well as dose and intensity should be considered when drawing conclusions.

## Introduction

PTSD is among the most prevalent mental disorders worldwide (Almeida et al., 2021). As a chronic psychiatric disorder that develops following exposure to a traumatic event, PTSD is a long-lasting and disabling condition that substantially impairs quality of life. Although the pathophysiology of PTSD is not fully known, alterations in the hypothalamic–pituitary–adrenal (HPA) axis, dysregulation of neurotransmitters and neurotrophins, and abnormalities in brain circuitry have been reported (Al Jowf et al., 2023).

BDNF is one of the most extensively studied neurotrophins in psychiatric disorders, including PTSD. BDNF is involved in the regulation of stress response and the effects of antidepressant treatment and has a complex role in PTSD pathophysiology (Notaras anf Van Den Buuse, 2020). It has been reported that patients with PTSD exhibit reduced plasma BDNF concentrations (Spudic et al., 2022). In addition to BDNF, Insulin-like growth factor-1 (IGF-1) is a potent neurotrophic factor that has been proposed as a potential biomarker for PSTD. IGF-1 has been demonstrated to exert anxiolytic effects and alleviate post-traumatic stress (Corne et al., 2021, Fernández De Sevilla et al., 2022).

Accumulating evidence suggests that sex hormones influence PTSD. Sex hormones influence the occurrence, symptoms, and clinical outcomes of PTSD (Hiscox et al., 2023, Henry et al., 2022, Velasco et al., 2023). For instance, it has been shown that PTSD-induced impairment in fear extinction memories is strongly modulated by gonadal hormones, however, the underlying mechanisms remain incompletely understood (Velasco et al., 2023).

According to existing studies, there is a reciprocal effect of glucocorticoids and sex hormones. Gonadal hormones are influenced by glucocorticoids, and the hypothalamic-pituitary-gonadal axis is also involved in regulating the HPA axis. Therefore, the HPA axis response to stress is affected by sex. This may explain disparities in the prevalence, severity, and response to treatment in stress-induced anxiety and depression between male and female individuals (Henry et al., 2022).

Exercise has been reported to have positive effects on mental health, including improving mood state and self-esteem and reducing stress and anxiety levels (Mikkelsen et al., 2017). It has therefore been proposed as a complementary intervention to reduce anxiety-related impairments. (Yakhkeshi et al., 2022, Shafia et al., 2017, Shafia et al., 2023b). However, exercise has a complex effects on stress hormones, as it leads to both a decrease and an increase in glucocorticoid levels (Wang et al., 2021, Yakhkeshi et al., 2022). According to some studies, exercise can induce a stress response depending on the exercise protocol. It has been reported that forced exercise increases ACTH and corticosterone levels and increases anxiety. Also, forced exercise fails to increase levels of BDNF (Griesbach et al., 2012, Svensson et al., 2016). Consistent with these findings, we found that our treadmill training protocol failed to improve some measures of anxiety-like behavior in ovariectomized rats exposed to SPS (Yakhkeshi et al., 2022). Therefore, the present study was designed to investigate whether a voluntary wheel-running exercise protocol produces greater therapeutic effects than a forced treadmill exercise protocol on PTSD-induced impairments exacerbated by ovariectomy. Specifically, we compared the effects of these two exercise protocols on corticosterone levels, neurotrophin expression, anxiety-like behavior, and spatial memory in an ovariectomized rat model of PTSD.

## Methods and materials

### Experimental animals

**Fig. 1:**
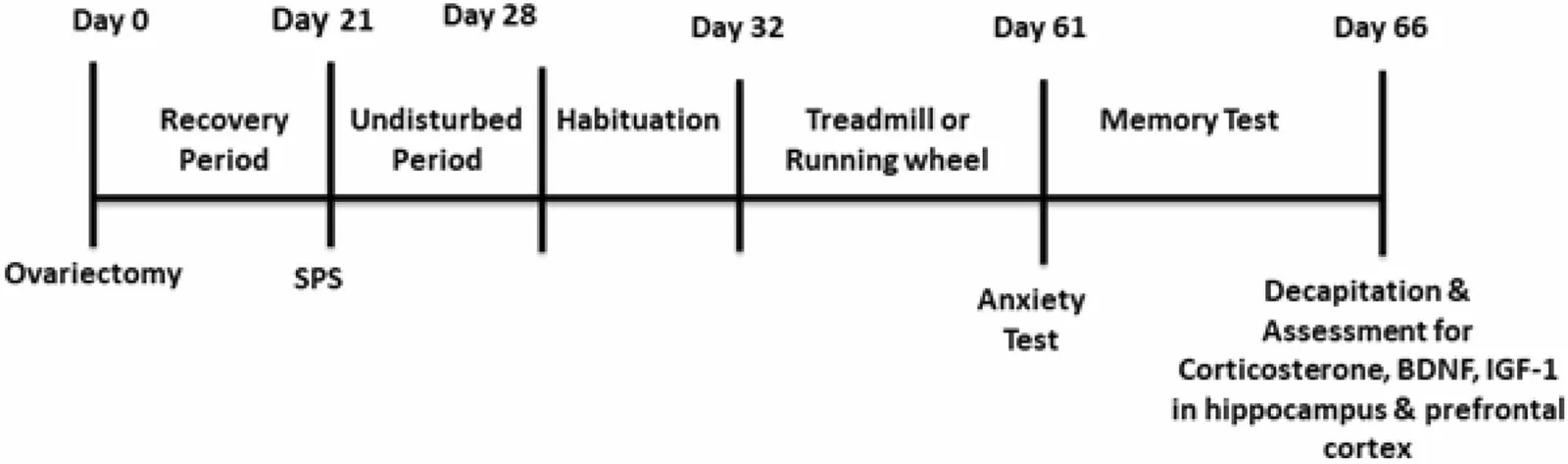
Timelines of experiments.

Female adult Wistar rats, aged 2.5–3 months, weighing between 200 and 250 g, were obtained from the animal research center of Mazandaran University of Medical Science, Sari, Iran. The animals were kept under standard conditions (temperature 26–28 °C, relative humidity 10–50% with 12/12 h light-dark cycle) and had access to water and food ad libitum. All experiments were performed in accordance with the research ethics committee and national guidelines for conducting animal studies. Female adult Wistar rats were randomly allocated to the control (CON), sham, and ovariectomized (OVX) groups. Following PTSD induction, animals within each group were randomly assigned to sedentary (SED), treadmill running (TR), or wheel running (RW) groups. The animals (n = 126) were allocated into 18 groups (each 7 rats) according to removal of ovaries, SPS induction and exercise protocol. Animals in control groups did not undergo ovariectomy while those in ovariectomy groups were subjected to a bilateral removal of ovaries surgery. In the sham group, after incision and exposing the ovaries, the skin and muscle layers were sutured without removing the ovaries. Regarding the exercise protocol, animals were divided into sedentary (SED), treadmill (TR), or wheel running (RW) groups. One animal from the OVX/SPS/TR group and one animal from the Sham/SPS/RW group were excluded because they failed to achieve the predefined minimum running distance. These animals were replaced to maintain the predetermined sample size, resulting in a final sample size of seven animals per group. The timeline of the research is shown in Fig. 1.

### Ovariectomy procedure

Animals were deeply anesthetized by an intraperitoneal injection of ketamine (100 mg/kg) and xylazine (2.5 mg/kg). After disinfecting the site, a small incision was made in the lateral abdominal wall bilaterally to remove the ovaries. The skin and muscle walls were then sutured. 20 days were assigned for surgical recovery and elimination of endogenous ovarian hormones.

### Single prolonged stress model

SPS, as one of the most prevalent animal models of PTSD, can help to study the neurobiological basis of PTSD and develop new treatment strategies (Török et al., 2019). The SPS was conducted in three stages: restraining for two hours, forced swimming for 20 min, and anesthesia by ether. The size of the restrainer should be appropriate for the animal that is restrained. After being restrained, the rats were forced to swim for 20 min in a cylindrical container 240 mm diameter and 500 mm high that was filled up with water to two-thirds of its height. After a 15-minutes recuperation period, the animals were anesthetized with diethyl ether (Liberzon et al., 1997) and then left undisturbed in their home cage for 7 days.

### Exercise training protocols

#### Treadmill training

After the ovariectomy and SPS, exercise started on day 32. Exercise was performed according to the protocol described in our previous study (Eshaghi-Gorji et al., 2024). For habituation to the treadmill, the rats walked on the treadmill for 15 min at the lowest speed (3 m/min) for three days. After being habituated, moderate exercise started for the treadmill exercise groups. The forced exercise group was forced to run on a treadmill with a 0-degree incline for 30 min at 10 m/min 5 days/week for 4 weeks. Rats in the sedentary groups were placed on the switched-off treadmill for 5 min once a day.

#### Wheel running training

The running wheel exercise was started on the 32nd day of the experiment. The rats, in the wheel running group, were individually given free access to a running wheel (circumference, 100 cm) attached to their cages for 24 h 4 weeks (Mazur et al., 2017). The running wheel apparatus was equipped with a revolutions counter. The running distances were monitored daily. The rats that ran less than 2500 m for 4 weeks were excluded from the study.

### Anxiety-like behavior assessment, Open Field Test

The open field apparatus consisted of an enclosed square arena made of dark opaque Plexiglas (60 × 60 cm) with walls 30 cm high. Painted white lines divided the area into 16 squares (15 ×15 cm each). The open field box was cleaned with alcohol thoroughly between sessions to assess anxiety-like responses. Rats were placed in the center of the open field box. Total movement was tracked for 5 min and analyzed for evaluation of anxiety behavior. Total distance traveled, time spent in the center of the arena, and time spent in the peripheral zone were measured to assess anxiety-like behavior (Ranjbar et al., 2018, Lee et al., 2016). Reduced time spent in the center of the arena was interpreted as decreased exploratory behavior and increased anxiety-like behavior (Ranjbar et al., 2018).

### Object location memory task (OLMT)

The novel object location task is a widely used behavioral test to evaluate spatial memory in rodents. Rodents tend to spend more time exploring the novel location than one that remains in a familiar location. The test is scored based on the differences in the exploration time of novel and familiar locations. The objects were placed in a square, black open-field (71 × 71 × 50 cm) under dim light (70 lx), 11 cm from the walls. The procedure consisted of habituation, familiarization, and test phases. In the habituation phase, the rats were habituated to the experimental apparatus by allowing them to explore it 10 min/day for three consecutive days without objects. In the familiarization phase, the rats were placed in the open-field arena and exposed to two identical sample objects (A + A) for 5 min. In the test phase on the next day, the rats were placed in the open-field arena where one of the objects was relocated to a novel position. A digital camera placed above the arena and connected to a videotape was used to track rat behavior during the exploration time. The time spent exploring the familiar and novel locations were recorded for 5 min. The exploration time was calculated as follows: time of exploration of novel location/ (time of exploration of familiar location + time of exploration of novel location). An exploration time equal to 50% reflects no preference for any of the locations. An exploration time percentage higher than 50% shows a preference for the novel location (Shafia et al., 2017).

### Measurement of BDNF and IGF-1 and corticosterone in hippocampal and prefrontal cortices

After behavior tests, under deep anesthesia, the rats were decapitated and the hippocampus and prefrontal cortices were quickly isolated and stored at − 80 °C until analysis day. Hippocampal and prefrontal cortical tissues were collected for biochemical analysis by ELISA at approximately 12:00 PM (noon) to minimize the effects of biological circadian variation, particularly in corticosteroid levels. To reduce the effects of acute stress induced by behavioral testing, brain tissue was collected approximately 1 h after completion of the final behavioral test for subsequent biochemical analyses, including corticosterone measurement (Spencer and Deak, 2017)

The cortices were cut into small pieces and homogenized in phosphate-buffered saline (PBS), pH 7.4 by the homogenizer and then centrifuged for 10 min at 10000 *g* at 4 ℃. The levels of BDNF and IGF-1 and corticosterone in hippocampus and prefrontal cortices were assayed by the E-Max ELISA method, according to the manufacturer’s instruction using Rat BDNF and IGF-1 and corticosterone ELISA kits (ZellBio GmbH Germany). The absorbance of samples was read at 450 nm by a micro plate reader (RT-2100C, Germany) and values calculated according to related standard curves. BDNF (the assay range 25 −800 pg/ml and the sensitivity of the assay was 3 pg/ml), and IGF-1 (the assay range 30 −960 ng/ml and the sensitivity of the assay was 4 ng/ml) and corticosterone (The intra and inter assay coefficients of variation were *<* 4.1% and *<* 6.4%, respectively and the sensitivity of the assay was *<*1.6 nm/l) (Eshaghi-Gorji et al., 2024, Shafia et al., 2023b).

### Statistical analysis

Descriptive statistics were expressed as Mean±SEM. The three-way ANOVA (Exercise × SPS× Ovariectomy) with Tukey's HSD post-hoc test was done to identify statistically significant differences. SPSS software (version 21) was used for data analysis and p < 0.05 was considered a significant level.

## Results

### Anxiety profile

#### Anxiety profile

##### Time spent in the central zone & Time spent in the peripheral zone

**Fig. 2:**
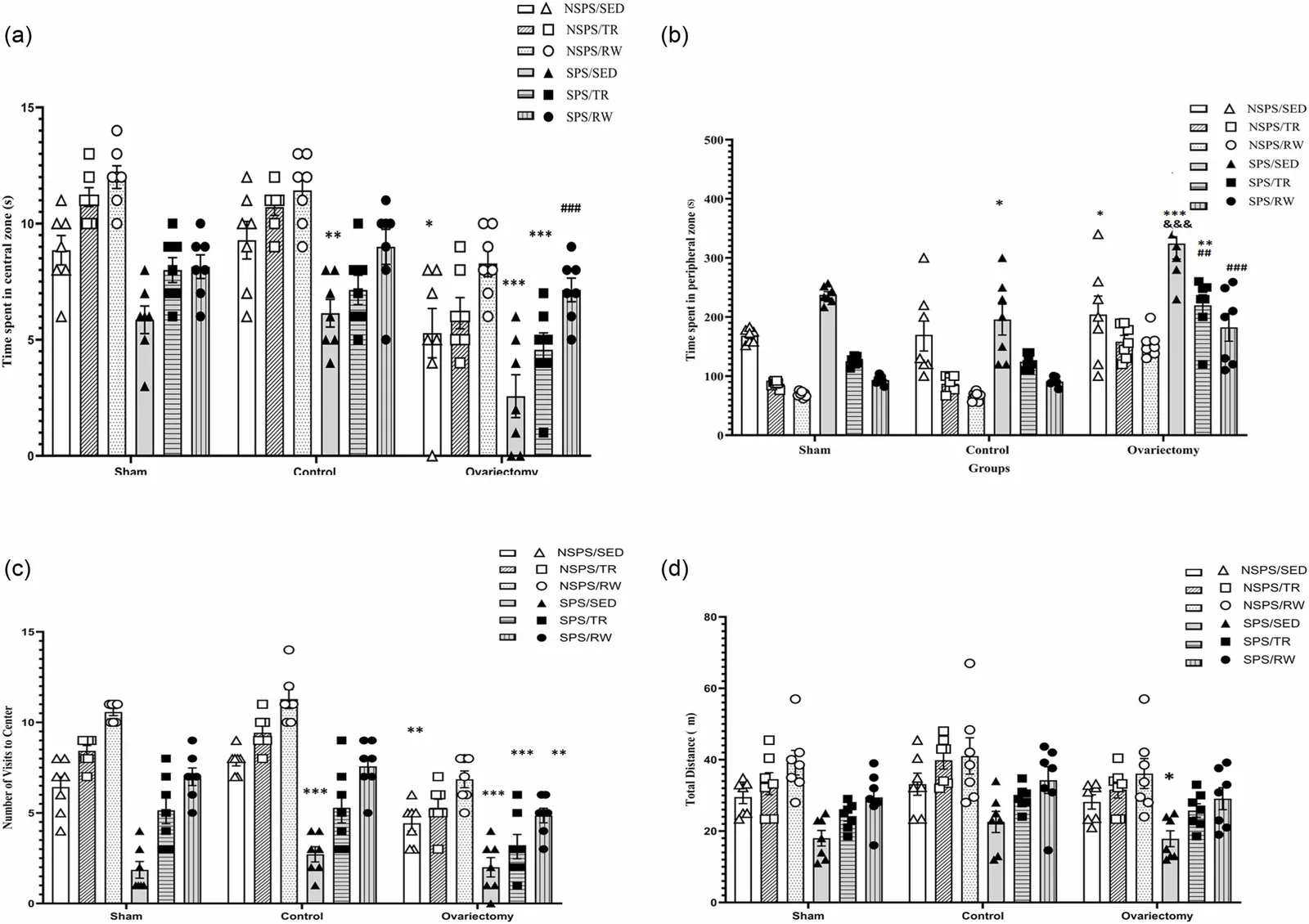
**A**. Time spent in central zone, assessed by the Open field, in SPS and ovariectomy rats subjected to treadmill and running wheel. * (P = 0/024), ** (P = 0/003), *** (P = 0/0001), significant difference with CON/NSPS/SED. # # # (P = 0/0001), significant difference with OVX/SPS/SED. **B**. Time spent in peripheral zone, assessed by the open field, in SPS and ovariectomy rats subjected to treadmill and running wheel, * (P = 0/042), (P = 0/015), ** (P = 0/002), *** (P = 0/0001), significant difference with CON/NSPS/SED. & & & (P = 0/0001), significant difference with CON/SPS/SED. # # (P = 0/002), # # # (P = 0/0001), significant difference with OVX/SPS/SED. **C**. Number of visit center, assessed by the Open field, in SPS and Ovariectomy rats subjected to treadmill and running wheel, *** (P = 0/0001), ** (P = 0/001), ** (P = 0/005), significant difference with CON/NSPS/SED. **D**. Total Distance, assessed by the Open field, in SPS and Ovariectomy rats subjected to treadmill and running wheel, * (P = 0/028) significant difference with CON/NSPS/SED.

The data for the time spent in the central zone during the open field test are presented in Fig. 2A. A three-way ANOVA revealed significant main effects of SPS (F₁,₁₀₈ = 77.378, P = 0.0001), ovariectomy (F₂,₁₀₈ = 50.542, P = 0.0001), and exercise (F₂,₁₀₈ = 31.208, P = 0.0001). The results of the post hoc between-group comparisons are presented in Fig. 2A.

The data for the time spent in the peripheral zone during the open field test are presented in Fig. 2B. A three-way ANOVA revealed significant main effects of SPS (F₁,₁₀₈ = 43.018, P = 0.0001), ovariectomy (F₂,₁₀₈ = 53.820, P = 0.0001), and exercise (F₂,₁₀₈ = 80.150, P = 0.0001), as well as a significant SPS × exercise interaction (F₂,₁₀₈ = 3.368, P = 0.038). The results of the post hoc between-group comparisons are presented in Fig. 2B**.**

##### Number of visits to the center & Total distance traveled in meters

The data for the number of visits to the center zone are presented in Fig. 2C. A three-way ANOVA revealed significant main effects of SPS (F₁,₁₀₈ = 121.61, P = 0.0001), ovariectomy (F₂,₁₀₈ = 98.028, P = 0.0001), and exercise (F₂,₁₀₈ = 43.98, P = 0.0001). A significant interaction between SPS and ovariectomy was also observed (F₂,₁₀₈ = 13.121, P = 0.001). The results of the post hoc between-group comparisons are presented in Fig. 2C.

The data for the total distance traveled (m) are presented in Fig. 2D. A three-way ANOVA revealed significant main effects of SPS (F₁,₁₀₈ = 23.63, P = 0.001), ovariectomy (F₂,₁₀₈ = 9.031, P = 0.004), and exercise (F₂,₁₀₈ = 10.350, P = 0.0001). The results of the post hoc between-group comparisons are presented in Fig. 2D.

##### Location memory test

**Fig. 3:**
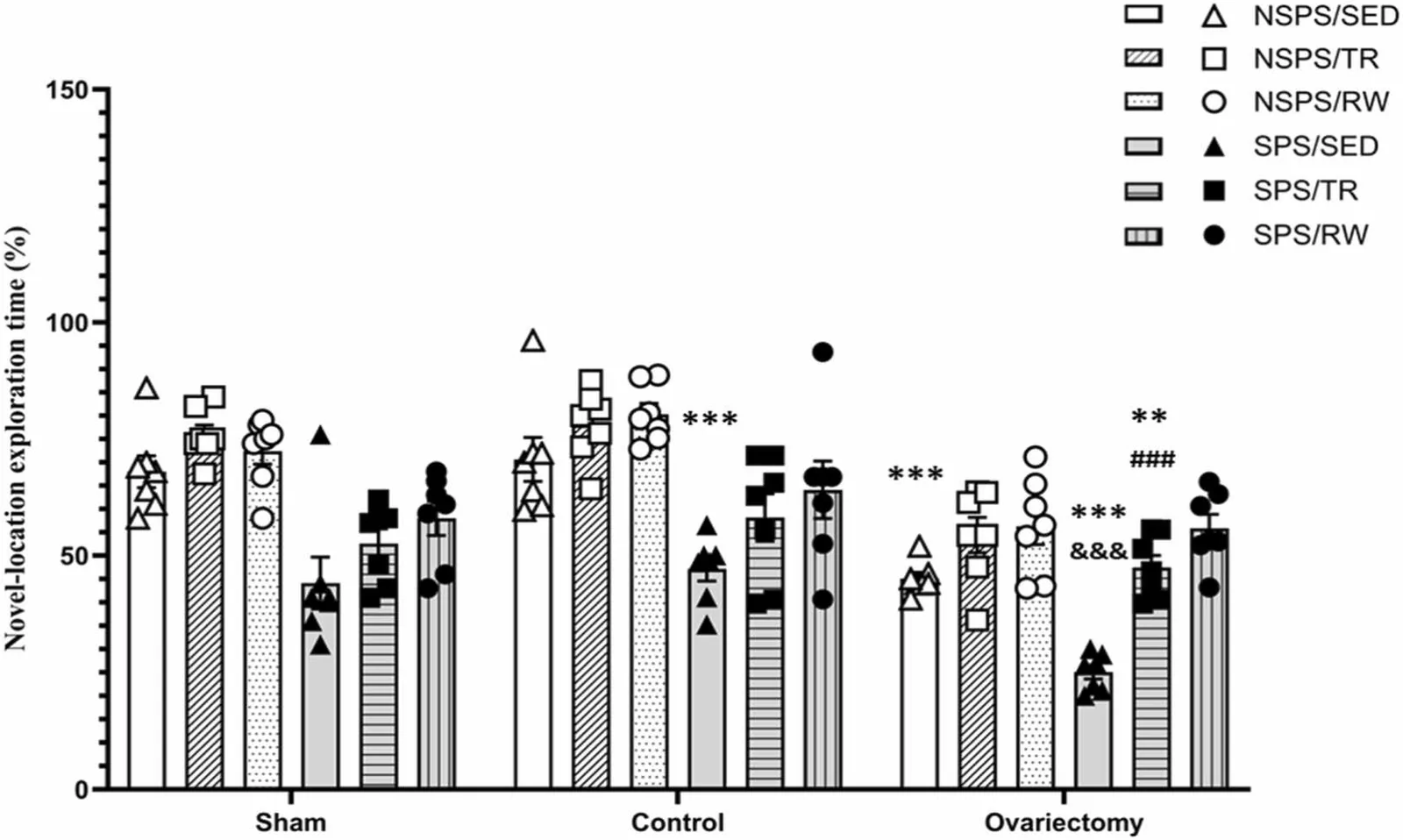
**Exploration time percentage, assessed by the location memory test, in SPS and ovariectomy rats subjected to treadmill and running wheel,** ** (P = 0/003), *** (P = 0/0001), significant difference with CON/NSPS/SED. & & & (P = 0/0001), significant difference with CON/SPS/SED. # # # (P = 0/0001), significant difference with OVX/SPS/SED.

The data for the exploration time percentage are presented in Fig. 3. A three-way ANOVA revealed significant main effects of SPS (F₁,₁₀₈ = 177.846, P = 0.0001), ovariectomy (F₂,₁₀₈ = 116.779, P = 0.0001), and exercise (F₂,₁₀₈ = 182.446, P = 0.0001). Significant SPS × ovariectomy (F₂,₁₀₈ = 7.135, P = 0.001) and SPS × exercise (F₂,₁₀₈ = 3.098, P = 0.049) interactions were also observed. The results of the post hoc between-group comparisons are presented in Fig. 3.

##### Hippocampal and prefrontal corticosterone levels

**Fig. 4:**
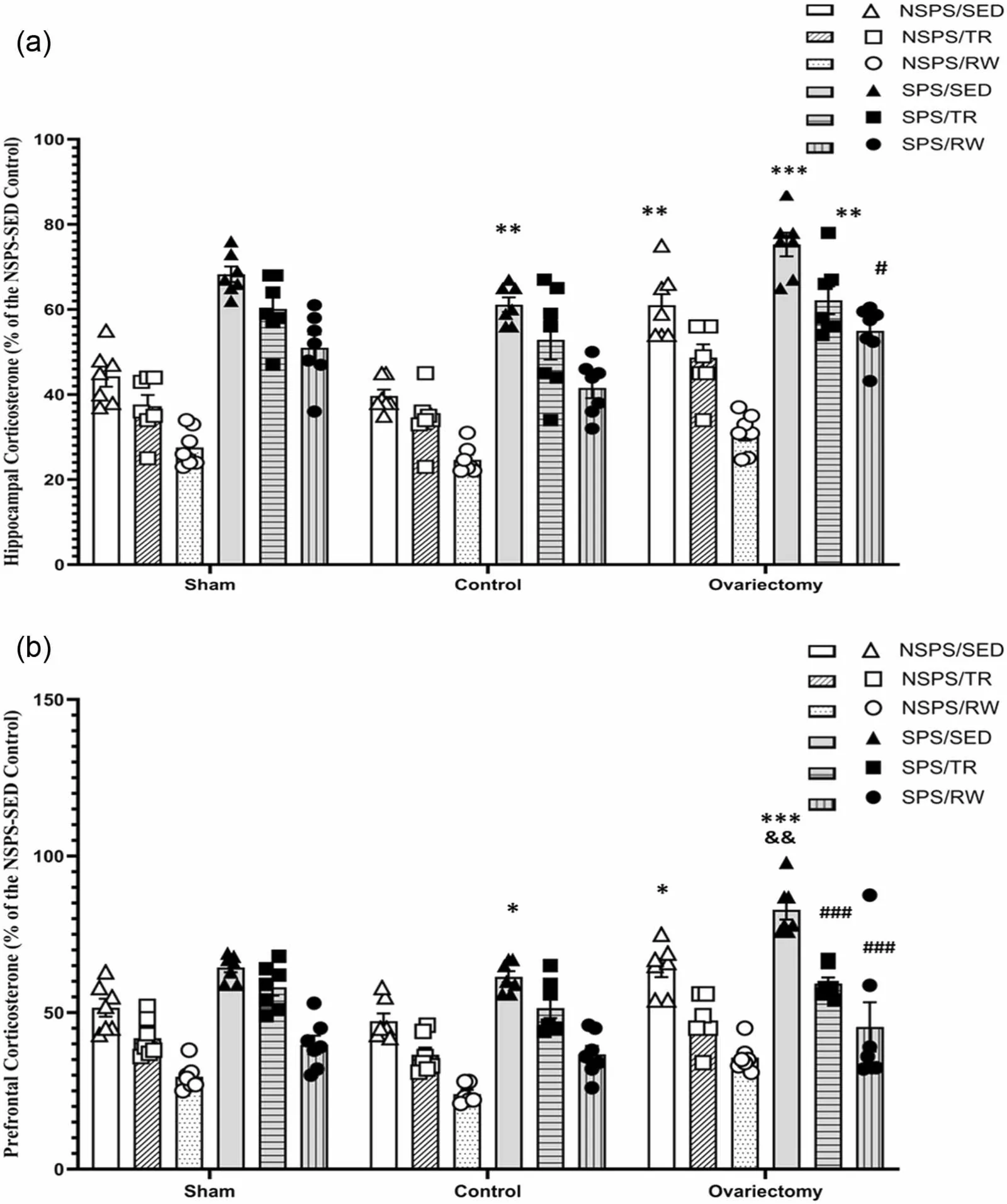
**A. Hippocampal corticosterone in SPS and ovariectomy rats subjected to treadmill and running wheel** ** (P = 0/005), ** (P = 0/006), ** (P = 0/003), *** (P = 0/0001) significant difference with CON/NSPS/SED, # (P = 0/010) significant difference with OVX/SPS/SED. **B. prefrontal corticosterone in SPS and ovariectomy rats subjected to treadmill and running wheel.** * (P = 0/012), * (P = 0/021), ** (P = 0/003), *** (P = 0/0001) significant difference with CON/NSPS/SED, & & (P = 0/001) significant difference with CON/NSPS/SED, # # # (P = 0/0001), significant difference with OVX/SPS/SED.

The data for hippocampal corticosterone levels are presented in Fig. 4A. A three-way ANOVA revealed significant main effects of SPS (F₁,₁₀₈ = 73.079, P = 0.0001), ovariectomy (F₂,₁₀₈ = 18.196, P = 0.0001), and exercise (F₂,₁₀₈ = 64.291, P = 0.0001). The results of the post hoc between-group comparisons are presented in Fig. 4A.

The data for prefrontal corticosterone levels are presented in Fig. 4B. A three-way ANOVA revealed significant main effects of SPS (F₁,₁₀₈ = 106.566, P = 0.0001), ovariectomy (F₂,₁₀₈ = 31.378, P = 0.0001), and exercise (F₂,₁₀₈ = 131.529, P = 0.0001). The results of the post hoc between-group comparisons are presented in Fig. 4B.

##### Hippocampal and prefrontal IGF-1 levels

**Fig. 5:**
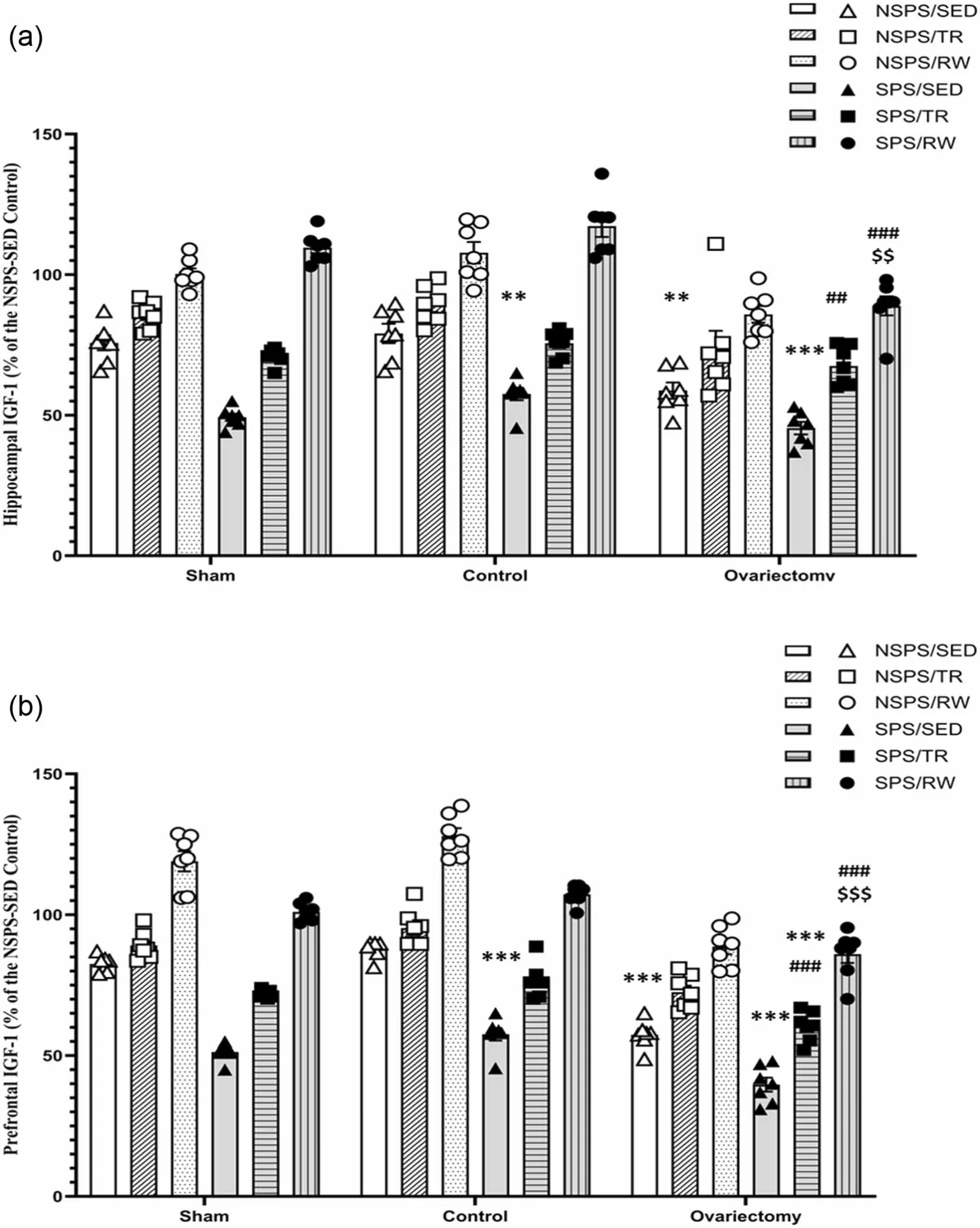
**A. Hippocampal IGF-1 in SPS and ovariectomy rats subjected to treadmill and running wheel,** ** (P = 0/002), ** (P = 0/004), *** (P = 0/0001 significant difference with CON/NSPS/SED, # # (P = 0/001), # # # (P = 0/0001), significant difference with OVX/SPS/SED. $ $ (P = 0/002) significant difference with OVX/SPS/TR. **B. Prefrontal IGF-1 in SPS and ovariectomy rats subjected to treadmill and running wheel,** *** (P = 0/0001) significant difference with CON/NSPS/SED, # # # (P = 0/0001), significant difference with OVX/SPS/SED. $ $ $ (P = 0/0001) significant difference with OVX/SPS/TR.

The data for hippocampal IGF-1 levels are presented in Fig. 5A. A three-way ANOVA revealed significant main effects of SPS (F₁,₁₀₈ = 27.845, P = 0.0001), ovariectomy (F₂,₁₀₈ = 57.390, P = 0.0001), and exercise (F₂,₁₀₈ = 271.676, P = 0.0001). Significant ovariectomy × exercise (F₄,₁₀₈ = 2.807, P = 0.029) and SPS × exercise (F₂,₁₀₈ = 30.198, P = 0.0001) interactions were also observed. The results of the post hoc between-group comparisons are presented in Fig. 5A.

The data for prefrontal IGF-1 levels are presented in Fig. 5B. A three-way ANOVA revealed significant main effects of SPS (F₁,₁₀₈ = 307.513, P = 0.0001), ovariectomy (F₂,₁₀₈ = 198.969, P = 0.0001), and exercise (F₂,₁₀₈ = 546.645, P = 0.0001). Significant ovariectomy × exercise (F₄,₁₀₈ = 2.778, P = 0.030), SPS × exercise (F₂,₁₀₈ = 13.547, P = 0.0001), and SPS × ovariectomy (F₂,₁₀₈ = 14.733, P = 0.0001) interactions were also observed. The results of the post hoc between-group comparisons are presented in Fig. 5B.

##### Hippocampal and prefrontal BDNF levels

**Fig. 6:**
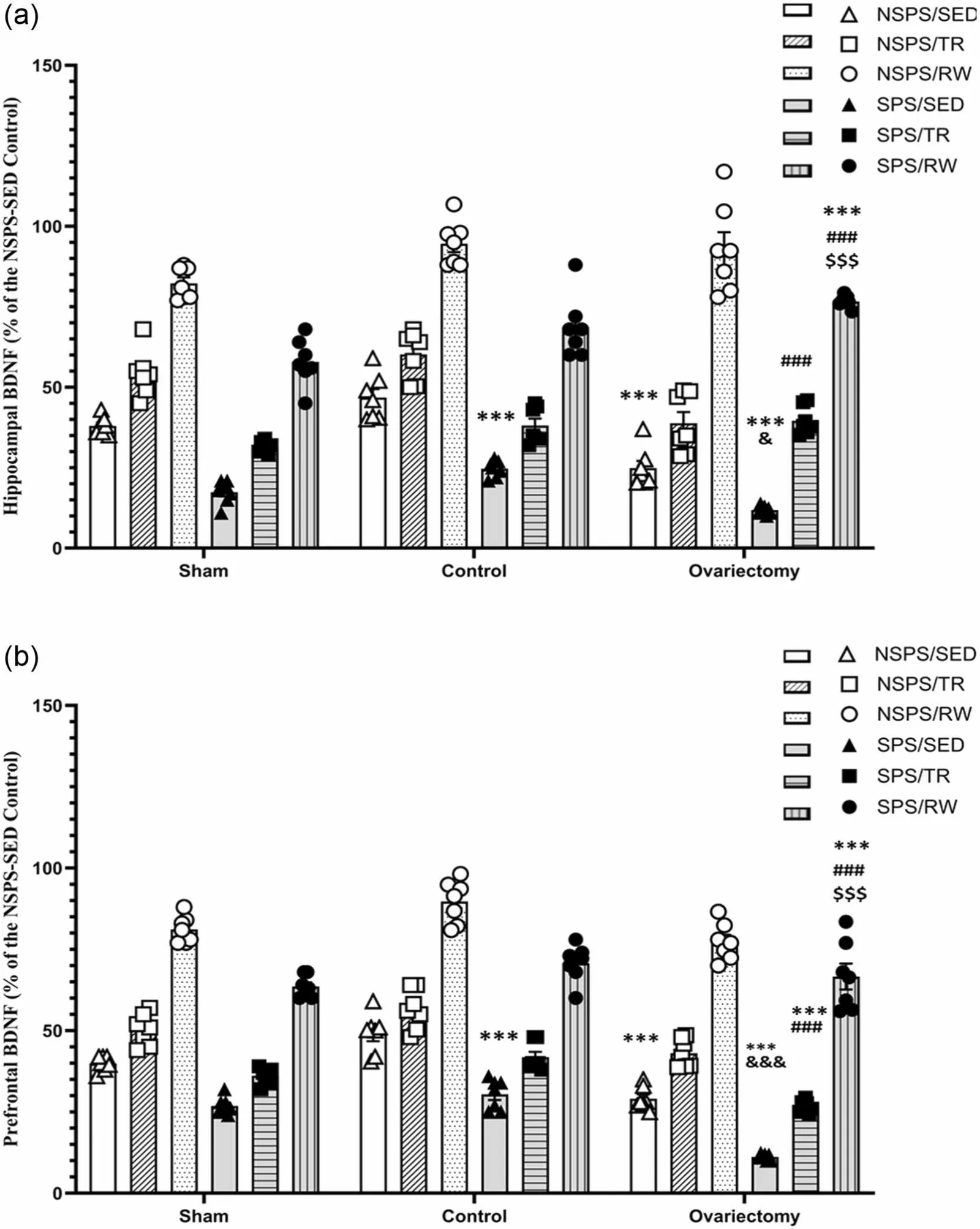
**A. Hippocampal BDNF in SPS and ovariectomy rats subjected to treadmill and running wheel,** *** (P = 0/0001) significant difference with CON/NSPS/SED, &(P = 0/043) significant difference with CON/NSPS/SED, # # # (P = 0/0001), significant difference with OVX/SPS/SED. $ $ $ (P = 0/0001) significant difference with OVX/SPS/TR. **B. Prefrontal BDNF in SPS and ovariectomy rats subjected to treadmill and running wheel,** *** (P = 0/0001) significant difference with CON/NSPS/SED, & & & (P = 0/0001) significant difference with CON/NSPS/SED, # # # (P = 0/0001), significant difference with OVX/SPS/SED. $ $ $ (P = 0/0001) significant difference with OVX/SPS/TR.

The data for hippocampal BDNF levels are presented in Fig. 6A. A three-way ANOVA revealed significant main effects of SPS (F₁,₁₀₈ = 97.884, P = 0.0001), ovariectomy (F₂,₁₀₈ = 4.844, P = 0.010), and exercise (F₂,₁₀₈ = 328.566, P = 0.0001). Significant SPS × exercise (F₂,₁₀₈ = 5.154, P = 0.007), ovariectomy × exercise (F₄,₁₀₈ = 8.304, P = 0.0001), and SPS × ovariectomy (F₂,₁₀₈ = 5.312, P = 0.006) interactions were also observed. The results of the post hoc between-group comparisons are presented in Fig. 6A.

The data for prefrontal BDNF levels are presented in Fig. 6B. A three-way ANOVA revealed significant main effects of SPS (F₁,₁₀₈ = 163.495, P = 0.0001), ovariectomy (F₂,₁₀₈ = 27.846, P = 0.0001), and exercise (F₂,₁₀₈ = 261.758, P = 0.0001). Significant ovariectomy × exercise (F₄,₁₀₈ = 5.067, P = 0.001), SPS × ovariectomy (F₂,₁₀₈ = 10.688, P = 0.0001), SPS × exercise (F₂,₁₀₈ = 18.220, P = 0.0001), and SPS × ovariectomy × exercise (F₄,₁₀₈ = 6.787, P = 0.0001) interactions were also observed. The results of the post hoc between-group comparisons are presented in Fig. 6B.

## Discussion

According to our previous study, there is a complicated interaction between OVX and SPS on anxiety behavior. We found that although treadmill exercise improved cellular responses in SPS ovariectomized rats, it showed no significant effect on anxiety-like behaviors in EPM test (Yakhkeshi et al., 2022). The present study revealed that the wheel-running exercise protocol was more effective than the treadmill exercise protocol in improving SPS-induced anxiety-like behavior in the open field test in ovariectomized rats.

According to our results, ovariectomy was associated with an exacerbation of the adverse effects of SPS on the HPA axis, BDNF and IGF-1 levels, as well as anxiety behavior. It is well known that sex hormones affect stress responses, with women being more susceptible to PTSD than men (Ravi et al., 2019). Previous studies have demonstrated a mutual relationship between sex hormones and stress hormones in anxiety-related mental illnesses. Chronic stress exposure and cortisol release may inhibit the secretion of sex hormones and induce a hypogonadal state. Specifically, the HPA axis activity has been shown to influence ovarian hormones in females (Hu et al., 2024). On the other hand, sex hormones may play an important role in HPA function and the secretion of stress hormones in PTSD (Briscione et al., 2017, Yakhkeshi et al., 2022). Previous studies have shown that ovariectomized rats exhibit elevated corticosterone levels following exposure to stress. Ovariectomy has also been associated with a significant reduction in the number of pyramidal neurons in the hippocampal CA3 region and decreased hippocampal BDNF mRNA expression. Furthermore, the combination of ovariectomy and restraint stress results in significant impairments in cognitive function(Takuma et al., 2007). Clinical studies indicate that in women with low estrogen levels, fear-potentiated startle is higher during extinction in the PTSD group compared with traumatized control women (Glover et al., 2012). Moreover, genetic and epigenetic studies support a role for estrogen in the pathophysiology of PTSD. In this regard, PTSD has been associated with methylation of CpG sites in the *HDAC4* gene, and studies in ovariectomized mice have demonstrated that estrogen influences *HDAC4* regulation and expression, suggesting an estrogen-dependent mechanism underlying PTSD susceptibility (Maddox et al., 2018). Collectively, these findings highlight the important contribution of ovarian hormones to stress-related neurobiological processes and underscore the need for greater emphasis on females in PTSD research.

Since deficiency of ovarian hormones, specifically estradiol, may result in elevated PTSD symptomatology, such as fear extinction deficits, and exaggerated alterations in PTSD-associated hormones and cytokines (Garcia et al., 2018, Yakhkeshi et al., 2022), it is predictable that the response to treatment may decrease in the ovariectomy plus SPS states. Consistent with this possibility, our treadmill exercise protocol was unable to ameliorate most SPS-induced anxiety-like behaviors in ovariectomized rats, potentially reflecting the combined impact of ovariectomy and SPS exposure. However, wheel-running protocol improved the majority of anxiety-like behaviors, except for the number of visits to the center.

### The wheel-running protocol was more effective than the treadmill protocol in reducing corticosterone levels in ovariectomized rats exposed to SPS

Although the underlying mechanisms of PTSD are still incompletely understood, some involved hormones and cytokines have been reported. (Fernández De Sevilla et al., 2022). In this regard, one of the most common pathophysiological findings is disruptions in the HPA axis and cortisol regulation (Fischer et al., 2021). Surprisingly, cortisol and corticosterone levels have been reported to be either increased or decreased in stress-related disorders (Sarapultsev et al., 2020). The difference in results of glucocorticoid levels after the PTSD model is supposed to be related to the difference in measuring time (Chen et al., 2020). Time-dependent change in cortisol levels after PTSD has also been highlighted in a review study by Steudte-Schmiedgen and colleagues (Steudte-Schmiedgen et al., 2016). In this regard, the Chen et al. study showed that the plasma level of corticosterone raised on the day 28th while it did not increase on the days 8th and 22nd (Chen et al., 2020). Correspondingly, in the present study, the corticosterone level in hippocampus & prefrontal cortex was high when it was measured 45 days after SPS induction. The increase in corticosterone levels in animal models of post-traumatic stress disorder has also been shown in other studies (Fenchel et al., 2015, Qiu et al., 2018, Eshaghi-Gorji et al., 2024).

The results showed that wheel-running protocol resulted in a greater reduction of the corticosterone level as compared to treadmill protocol. The different findings between voluntary wheel running and forced treadmill exercises can partly be explained by the fact that forced exercise can be a stressful experience and consequently can induce the rise of endogenous glucocorticoid production due to stress (Eldomiaty et al., 2020). Exercise, even forced treadmill exercise, could improve the disturbed HPA axis induced by SPS and ovariectomy. However, voluntary wheel running restored corticosterone levels to the normal range.

### The wheel-running protocol was more effective than the treadmill protocol in increasing IGF-1 concentrations in ovariectomized rats exposed to SPS

IGF-1 has been reported to possess anxiolytic properties and ameliorate PTSD-like behaviors in an animal model of PTSD (Fernández De Sevilla et al., 2022), hence its alteration is important in PTSD studies. Despite the role of IGF-1 in PTSD, studies investigating IGF-1 concentrations in PTSD patients are not satisfactory, and the limited findings are conflicting (Rusch et al., 2015). According to existing studies, stress states such as SPS or prenatal stress lead to decrease in IGF-1 (Shafia et al., 2023b, Basta-Kaim et al., 2014). In the present study, the diminution in IGF-1 following SPS induction may be attributed to glucocorticoid enhancement. In agreement to this statement, it has been reported that there is a negative correlation between serum glucocorticoid level and tissue IGF-1 expression as “glucocorticoid-insulin-like growth factor 1 (GC-IGF-1) axis”(He et al., 2019). Also, it has been reported that the administration of the potent glucocorticoid inhibits IGF-1 gene expression (Inder et al., 2010).

The present study demonstrated that aside from SPS, ovariectomy can individually reduce IGF-1 in the hippocampus and prefrontal cortex. The decrease in IGF-1 levels in the brain after ovariectomy has also been reported in Habibi and colleagues' study (Habibi et al., 2017). Because of the interaction between IGF-1 and female hormones in brain function (Bianchi et al., 2017, Baumgartner et al., 2022), the decrease in IGF-1 following ovariectomy may lead to more complicated treatment in SPS rats.

The present study found that both voluntary wheel running and forced treadmill were able to increase IGF-l levels impaired by SPS and ovariectomy, however, wheel-running protocol resulted in a higher level of IGF-1 than treadmill exercise. Indeed voluntary wheel running was able to restored IGF-1 levels toward the control range. Correspondingly, other studies have reported that the effects on IGF-1 differ depending on the type of exercise. For example, Chen and colleagues showed that IGF-1 levels in different exercise groups were superior to the control group, however, resistance training resulted in more increase in the level of IGF-1 (Chen et al., 2017). Interestingly, Kim and colleagues found that intense walking exercise reduced serum levels of IGF-1(Kim et al., 2015). Since forced exercise increases anxiety levels in females (Uysal et al., 2015), We presume that the complex relationships between forced exercise and anxiety levels in the female sex (Uysal et al., 2015) and between stress and glucocorticoid hormone levels (Sarapultsev et al., 2020) as well as between glucocorticoid and IGF-1 levels (He et al., 2019) likely led to different effect of forced treadmill and voluntary wheel running protocols in terms of increasing IGF-l. Since BDNF and IGF-I have been documented to play a key role in mediating the effects of exercise on brain structure and function (Maass et al., 2016), their increase after exercise may help predict whether the selected exercise was effective.

### The wheel-running protocol was more effective than the treadmill protocol in increasing BDNF levels in ovariectomized rats exposed to SPS

**Diagram 1:**
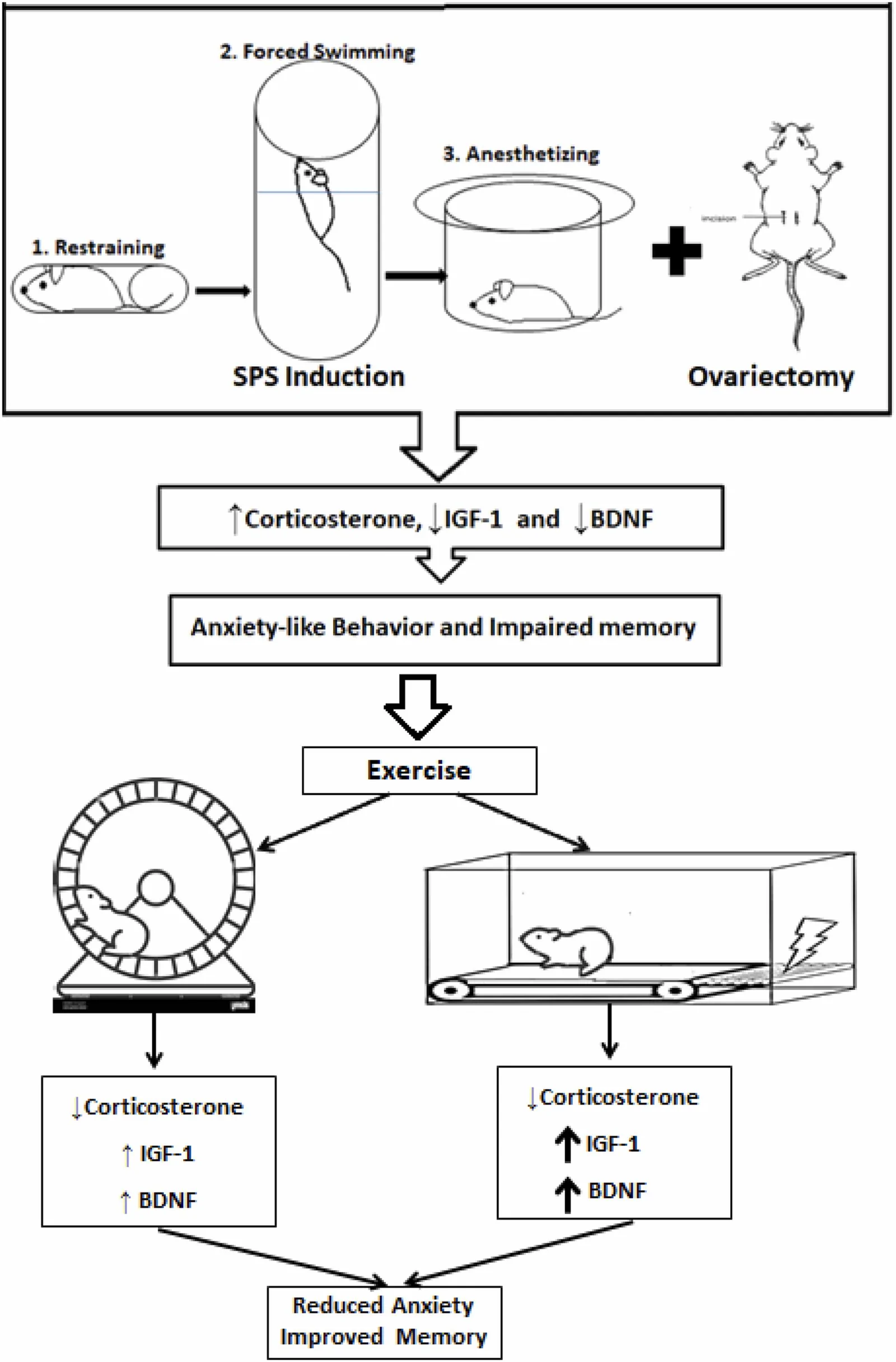
; An abstract of some factors involved in the induction of anxiety-like behavior and impairment in memory after SPS and the effects of two protocols of exercise on this factors.

In addition to glucocorticoid stress hormones, BDNF has been extensively investigated in psychiatric disorders, including PTSD (see the meta-analysis by Mojtabavi et al.)(Mojtabavi et al., 2020). According to existing studies, there is an interaction between glucocorticoids and BDNF in stress-related disorders. BDNF has been shown to play a regulatory role in the signaling of stress hormones in the brain, or to serve as the target of glucocorticoid signaling (Notaras anf Van Den Buuse, 2020). It is well known that increases in the glucocorticoid stress hormones decrease BDNF levels (Griesbach et al., 2012). Correspondingly, our study showed that after SPS induction, corticosterone increased while BDNF decreased. In addition to the effects of glucocorticoid hormones on BDNF, the negative effect of glucocorticoid hormones on IGF-I (He et al., 2019) and the correlation between IGF-I and BDNF have also been reported (Tonoli et al., 2015, Kazanis et al., 2004). Moreover, both BDNF and IGF-I have been shown to change in PTSD (Shafia et al., 2023a, Basta-Kaim et al., 2014) and the BDNF/IGF-1 system has been suggested to play a potential protective role in psychiatric disorders following stressors (Rusch et al., 2015). Based on these findings, a brief summary of possible relationships between corticosterone, BDNF, IGF-I, and anxiety-like behavior after SPS induction has been presented in schematic Diagram 1.

The present study revealed that despite the potential for both protocols to increase BDNF concentrations, the impact of wheel-running protocol was more pronounced. Since BDNF has been documented to play a key role in mediating the effects of exercise on brain structure and function (Maass et al., 2016), its greater increase following the wheel-running protocol may contribute to a greater therapeutic effect by ameliorating the brain dysfunction and dysregulation associated with SPS exposure and ovariectomy. Consistent with this possibility, the wheel-running group showed greater improvement in anxiety-like behaviors.

The difference between voluntary wheel running exercise and forced treadmill exercise in terms of the amount of BDNF rise has also been reported in Uysal and colleagues' study. They showed that forced treadmill exercise led to an increase in anxiety and a decrease in BDNF in adolescent female rats, whereas voluntary wheel running exercise led to the opposite outcomes (Uysal et al., 2015).

### The wheel-running protocol improved anxiety-like behavior in ovariectomized rats exposed to SPS

SPS is an experimental model in rodents that is capable of inducing the hallmark symptoms of PTSD, including fear and anxiety (Mancini et al., 2021). The present study showed that both protocols reduced SPS-induced anxiety-like behavior assessed by open field test. According to our results, SPS exposure was associated with changes in corticosterone, IGF-1, and BDNF levels, as well as alterations in anxiety-like behavior (schematic Diagram 1). As previously mentioned, exercise was able to restore corticosterone and IGF-1 to their normal ranges and increased BDNF levels and wheel-running protocol had a more significant effect than treadmill exercise on these factors. Therefore wheel-running protocol may improve anxiety-like behavior through potential mechanisms associated with the normalization of HPA axis activity and the increased expression of neurotrophic factors. Our findings are consistent with previous studies reporting anxiolytic effects associated with BDNF (Yin et al., 2022), stress-induced alterations in IGF-1 signaling (Basta-Kaim et al., 2014), and antidepressant-like effects of IGF-1 (Mueller et al., 2018). In this regard, BDNF has been reported to exert anxiolytic effects by attenuating the SPS-induced increase in tyrosine kinase receptor B (TrkB) protein and mRNA expression in the prefrontal cortex (PFC) and amygdala (Yin et al., 2022). In addition, Basta-Kaim et al. (2014) demonstrated that prenatal stress disrupts IGF-1 signaling by reducing IGF-1 levels and IGF-1 receptor phosphorylation, together with altered IRS-1 phosphorylation in the hippocampus and frontal cortex. These molecular alterations may contribute to the behavioral changes observed following prenatal stress (Basta-Kaim et al., 2014). Furthermore, Mueller et al. (2018) showed that infusion of IGF-1 into the medial prefrontal cortex produced antidepressant-like effects through activation of the mechanistic target of rapamycin complex 1 (mTORC1) (Mueller et al., 2018). Collectively, these studies provide biological plausibility for the associations observed in the present study, although further mechanistic investigations are required to determine whether BDNF and IGF-1 directly mediate the behavioral effects of exercise.

The current study found positive effects of both forced treadmill exercise and voluntary wheel- running exercise on anxiety-like behavior. However, studies have shown that forced exercise is a stressful experience in itself and may increase anxiety-like behaviors, especially in the female sex (Uysal et al., 2015, Leasure and Jones, 2008). Therefore, it is not surprising that the wheel- running exercise effect was greater than the treadmill exercise.

### Both wheel-running and treadmill exercise improved memory in ovariectomized rats exposed to SPS

Impaired learning and memory are characteristic hallmarks of post-traumatic stress disorder, and it has been demonstrated that SPS as an animal model of PTSD can induce decreased learning and memory in rats (Wen et al., 2017). Also, the Object Location Memory Test (OLMT), a commonly used behavioral assay for investigating learning and memory in rodents (Lueptow, 2017), can be used to examine memory impairment induced by SPS (Wen et al., 2017).

In the present study, both wheel running and treadmill protocols improved memory impairment induced by SPS and exacerbated by ovariectomy. The beneficial effect of exercise on memory and learning has been frequently reported in healthy subjects (see the review articles by Loprinzi and colleagues (Loprinzi et al., 2018) and Griebler and colleagues (Griebler et al., 2022). There are also some studies in PTSD patients or animal models of PTSD that showed the therapeutic effects of exercise on memory impairment (Koyuncuoğlu et al., 2021, Seo et al., 2019, Narita-Ohtaki et al., 2018). Furthermore, similar to our results, some studies have demonstrated that forced and voluntary exercises are equally effective in improving cognitive and memory impairments (Lin et al., 2015a, Belviranlı and Okudan, 2019, Lin et al., 2015b).

Since BDNF plays a crucial role in learning and memory by increasing synaptic plasticity (Powers et al., 2015), the increase in BDNF following both types of exercise may have contributed to the improvement in memory. In this regard, the mediating role of BDNF in the exercise-memory link has been reviewed by Loprinzi and Frith (Loprinzi and Frith, 2019). In addition to BDNF, it is reported that increased IGF-1 following exercise is associated with improved memory (Setiawan et al., 2024).

## Conclusion

Ovariectomy may exacerbate PTSD-related impairments, suggesting that ovarian hormone loss could influence the response to therapeutic interventions. In the present study, both the forced treadmill exercise protocol and the voluntary wheel running protocol improved behavioral and biochemical alterations induced by SPS in ovariectomized rats. However, the wheel-running protocol generally produced greater improvements than the treadmill protocol, particularly in anxiety-like behavior and neurobiological markers. These findings suggest that the voluntary wheel-running protocol may be a promising non-pharmacological strategy for alleviating PTSD-related impairments following ovariectomy. Nevertheless, because the treadmill and wheel-running protocols differed in exercise dose, intensity, and stress load, these factors should be carefully considered when interpreting the comparative effects of the two exercise paradigms. Further studies using dose-matched exercise protocols and mechanistic approaches are needed to confirm these findings and determine their translational relevance to women with PTSD.

## Strengths and limitations of study

Although bilateral ovariectomy is a well-established animal model for inducing ovarian hormone deficiency, the assessment of serum estradiol levels, uterine weight, vaginal cytology, or other physiological markers would have strengthened the validation of estrogen deficiency in the present study. Using animal models allows researchers to obtain essential findings that cannot be ethically or practically investigated in humans, such as detailed histological analyses. Although animal models studies are important tools for understanding the cellular and molecular mechanisms underlying diseases, as well as the histological changes and gene expression alterations in specific regions of the brain, behavioral scientists remain concerned about the translational validity of animal studies of mental disorders, such as PTSD, to humans.

## Availability of data and materials

The datasets used and/or analyzed during the current study are available from the corresponding author on reasonable request.

## CRediT authorship contribution statement

**Sakineh Shafia:** Supervision, Funding acquisition, Conceptualization. **Kobra Akhoundzadeh:** Writing – review & editing, Writing – original draft. **Seyed Parsa Seyedpour:** Investigation. **Seyedeh rose Jamali:** Methodology, Investigation. **Ali Shushtari:** Methodology, Investigation. **Maryam Janbazi Maryam:** Investigation. **Arvin Amiri:** Investigation.

## Ethical approval

This study was approved by the Iran National Committee for Ethics in Biomedical Research (IR.MAZUMS. AEC.1402.053)

## Funding

This work was supported by grants from the Mazandaran University of Medical Sciences, Sari, Iran, record no: 17716.

## Declaration of Competing Interest

The authors have no conflicts of interest to disclose that could influence this work.
